# Towards identification of true cancer biomarkers

**DOI:** 10.1186/s12916-014-0156-8

**Published:** 2014-09-11

**Authors:** Eleftherios P Diamandis

**Affiliations:** Department of Laboratory Medicine and Pathobiology, University of Toronto, Toronto, Ontario Canada; Department of Pathology and Laboratory Medicine, Mount Sinai Hospital, Toronto, Ontario Canada; Department of Clinical Biochemistry, University Health Network, Toronto, Ontario Canada; Mount Sinai Hospital, Joseph & Wolf Lebovic Ctr. 60 Murray St. (Box 32); Floor 6, Room L6-201, Toronto, ON M5T 3 L9 Canada

**Keywords:** Cancer biomarker, Genomics, Proteomics, Mutant proteins, High specificity, Selected reaction monitoring, Mass spectrometry

## Abstract

**Background:**

Most of newly discovered cancer biomarkers fail in the clinic because they lack sensitivity and/or specificity. The current explosion in knowledge of the mutational spectrum of many cancer types, as a result of whole exome and whole genome sequencing, has revealed a wide spectrum of mutations that appear to be highly specific for various cancer types.

**Discussion:**

Mass spectrometry (MS) has the ability to monitor tryptic peptides in complex biological mixtures with high sensitivity and specificity. It may be possible in the near future to combine the known spectrum of gene mutations revealed by genomics with the power of MS, in order to quantify mutant peptides that are highly specific for cancer, in a multiplex fashion. Such mutant peptides, quantified in the circulation and other fluids, may represent tumor markers that are suitable for detection and monitoring of cancer.

**Summary:**

The power of genomic and proteomic technologies can be combined to identify highly specific analytes for biomarker applications.

## Background

The National Institutes of Health define biomarkers as cellular, biochemical, and/or molecular (including genetic and epigenetic) characteristics by which normal and/or abnormal processes can be recognized and/or monitored. Biomarkers are measurable in biological materials, such as in tissues, cells, and/or bodily fluids. There are many examples of powerful biomarkers that are currently being used in clinical practice (for example, troponin, glucose, creatinine, and thyroid stimulating hormone, to mention only a few). Unfortunately, the current situation with cancer biomarkers is not so bright. Despite the fact that a handful of biomarkers are currently used in the clinic, none of them is useful for the highest-impact applications of population screening and early diagnosis. Currently, cancer biomarkers are deployed to aid in assessing therapeutic response in patients with advanced cancer or to provide earlier detection of relapse in patients who have been treated [1]. The main reason for their restricted use is that current cancer biomarkers are not cancer-specific proteins that are implicated in cancer initiation or progression, but rather molecules that are found in both normal and cancerous tissues, sometimes in roughly the same amounts. As would be expected, none of these markers is highly specific for cancer, and they are usually elevated in both malignant and non-malignant processes of the organs/cells that produce them.

Recently, it has been postulated that the emergence of highly powerful “omics” technologies, such as genomics, epigenomics, transcriptomics, proteomics, and metabolomics, may revolutionize the discovery of novel cancer biomarkers and/or panels, with distinct advantages over the currently used biomarkers [2]. However, this promise has not yet been fulfilled. Some of the reasons for the difficulties in discovering novel and improved cancer biomarkers have been discussed elsewhere [3,4]. In short, most of the newly discovered biomarkers either represent false discoveries, or are characterized by specificity that is similar, or more frequently, inferior to that of the currently used cancer biomarkers.

## Discussion

### Cancer heterogeneity

More recently, it has been recognized that cancer is a highly heterogeneous disease [5,6]. For example, several forms of epithelial ovarian cancer are currently recognized: high-grade serous (approximately 65% of cases), endometrioid (approximately 15%), clear cell (approximately 10%), low-grade serous (approximately 5%), and mucinous (approximately 5%) carcinoma. The mutational landscapes of these types of epithelial ovarian cancers are very different, with the serous histotype characterized by very high frequency of *TP53* mutations (96%) [7], and other subtypes having *TP5*3 mutations much less frequently (>30%) but having more frequent mutations in other genes (data from the Broad Institute’s tumor portal [8]). It is thus not surprising, given this information, that finding one universal cancer biomarker for all subtypes of epithelial ovarian cancer will be highly unlikely.

### Cancer-specific biomarkers

Do molecules that can only be found in cancer cells exist, and can they be identified? Such molecules could be highly specific markers for cancer and, if sufficiently sensitive, could represent the new generation of clinically useful biomarkers. Although some such cancer biomarkers have been identified (for example, translocation of parts of chromosomes 9 and 22 in chronic myelogenous leukemia, which creates an oncogenic *BCR-ABL* gene fusion; and some other translocations in rare cancers) these are the exceptions. For the most common forms of cancer, we do not as yet have cancer-specific molecules that can be used as biomarkers. Recently, I proposed that it may be possible in the future to identify “rare” tumor markers that occur in a small percentage (possibly 2 to 5%) of patients but have extremely high specificity (close to 100%) [9]. Here, I postulate that the new advances in genomics provide the opportunity to identify true cancer biomarkers. This may represent a possible solution to the stagnation of the cancer biomarker field over the past 30 years [10].

### Genomic alterations in cancer

Recently, Lawrence *et al*. analyzed the mutational spectrum of 21 cancer types within 4,742 specimens for which the exomes had been sequenced. This vast amount of new genomic data revealed that the number of significantly mutated genes (those present in >2% of tumors) in the 21 cancer types varied tremendously, from 1 to 58, with a mean of approximately 20. The Cancer Genome Atlas Research Network published the exomic sequencing of 316 serous ovarian cancers, and identified over 300 mutations, mostly affecting the *TP53* gene [7]. An important point made by Lawrence *et al*. is that the mutations so far identified represent an incomplete list. They calculated that in order to catalogue nearly all mutations found in human cancers, it will be necessary to sequence 600 to 5,000 tumors for each cancer type.

How could we translate this information into clinically useful biomarkers? Kandoth *et al*. showed that around 127 genes were significantly mutated in a combined analysis of 3,281 tumors, representing 12 tumor types [11,12]. Of these genes, a few (such as *BAP1*, *DNMT3A*, *KDM5C*, *FBX7* and *TP53*) were associated with poor prognosis, whereas mutations in two genes (*BRCA2* and *IDH1*) correlated with favorable prognosis. Thus, some mutated genes are progrnostic markers. Kinde *et al*., in an effort to translate some of the genomic findings into clinical diagnostic tools, collected Pap smears from patients with endometrial and ovarian cancers, and analyzed DNA from these samples to find mutations that are known to be associated with these cancers [13]. They could identify at least one mutation in all 24 endometrial cancers and in 40% (9 of 22) of ovarian cancers. Mutational analysis was performed with a highly sensitive and specific assay (Safe-Sequencing System; Safe-Seq S) which is immune to the presence of a vast excess of normal alleles [14]. An important finding of this study is that the specificity of the test was 100% in both cases, although the sample size was small (n = 14).

### Proteogenomics

Taking this one step further, it is reasonable to speculate that the encoded mutated proteins may represent the long-sought, highly specific cancer biomarkers of the future. To exemplify this point in some detail, I will use one example of the mutational spectrum reported by Kinde *et al*. for ovarian cancer [13]. The same concept can be applied to the myriad recently revealed mutations in other genes or other cancer types [11].

One mutation in the *TP53* gene reported by Kinde *et al*. is the V147D missense mutation, found in one out of the twenty-two ovarian cancers. Examination of tryptic peptides from p53 revealed that the normal allele will produce the tryptic peptide TCPVQLW**V**DSTPPPGTR, while the mutant allele will produce the peptide TCPVQLW**D**DSTPPPGTR (bold type indicates amino acid change). Examination of SRM Atlas [15] and GPM Global Proteome Machine [16] revealed that this peptide is present in both databases, and has a predicted m/z ratio of 955.9 as a doubly positively charged ion. Thus, development of a sensitive selected reaction monitoring (SRM) assay for both the wild-type and mutant peptides should be feasible.

Why would a proteomic/mass spectrometry (MS) method be advantageous over genomic approaches for identifying these mutations? First, it may be possible to identify mutated proteins that are secreted or membrane-bound, with the expectation that these proteins may be circulating and thus be accessible for analysis in blood. This may not be the case with genomic analysis, which requires tumor-specific DNA in the circulation. Second, at least theoretically, the mutated peptide should represent a unique fragment not present in normal cells (that is, a “true” cancer-specific biomarker). Third, the expected different fragmentation patterns of the wild-type and mutant peptides (due to a different amino acid in the mutated position) suggests that SRM analysis of the mutant peptide should be free from any interference by the wild-type peptide, even if the latter is present in huge excess. This speculation needs experimental verification.

As mentioned previously [9], it may be possible to utilize rare tumor markers (such as the ones mentioned above) for diagnosis and monitoring of those patients who are informative for this biomarker, despite low sensitivity (but excellent specificity). The vast number of mutations that have been revealed for ovarian and other cancers provide opportunities for combining rare, but highly specific, mutant peptides into a panel to improve sensitivity, while retaining outstanding specificity. The SRM assay is amenable to multiplexing, and more than 500 peptides can be monitored simultaneously with multiple transitions [17,18]. Thus, monitoring in parallel many peptides in one sample is highly realistic.

### Shortcomings

What are the possible shortcomings of such a method and ways around them? At the technical level, additional advances in MS will allow even greater multiplexing capability and higher sensitivity in detecting peptide fragments in SRM assays, in the presence of vast amounts of other peptides. It is also possible that some recently developed sample preparation protocols, such as stable isotope standard capture with anti-peptide antibodies (SISCAPA) [19] and others [20–22] may further help in enriching for the monitored peptides. It should also be expected that not all genetic mutations will be associated with tryptic peptides that have high ionization efficiency and can thus be detected by SRM. Moreover, the m/z ratio of these peptides may fall outside the dynamic range of current instruments. Another important caveat would be the abundance of the mutant peptide in the sample of interest, in comparison with the total amount of peptides in the tryptic mixture. Ion suppression may not allow efficient ionization of the peptide of interest. The detection of a few copies of a mutant peptide in a highly complex mixture such as a serum digest is likely to be a daunting task [23]. Underexpressed proteins will be even more difficult to quantify.

At the biological level, it may well be that many of these mutations are rare, or exist in proteins that are nuclear or cytoplasmic and thus not present in the circulation. In such cases, an easily accessible biological fluid may be a good substitute. Examples would be Pap smears and cervicovaginal fluid for endometrial, cervical, and ovarian cancer; sputum and bronchoalveolar lavage for oral and lung cancer, urine for bladder and prostate cancer; seminal plasma for prostate and testicular cancer; nipple aspirate fluid for breast cancer; and pancreatic juice for pancreatic cancer. Last, but not least, it should be emphasized that the identified mutations in cancer may not be oncogenic or even specific for cancer. Thus, the specificity of this approach needs to be experimentally determined.

## Summary

The majority of new biomarkers fail because they lack the necessary sensitivity and specificity to address an unmet clinical need. Our current ability to sequence tumor genomes provided a wide spectrum of mutations in many cancer types, which appear to be highly specific for cancer. Current advances in mass spectrometry allow for highly sensitive and specific monitoring of proteotypic peptides in complex biological mixtures. I speculate here that by combining the known mutational spectrum of genes, with the ability of MS to quantify mutant peptides in the presence of vast amounts of normal peptides in a multiplex fashion, we may be able to develop cancer-specific assays. It remains to be seen if this combination can lead to cancer biomarkers that have better characteristics than the ones currently used in the clinic.
